# Defects, Dopants and Lithium Mobility in Li_**9**_**V**_**3**_**(P**_**2**_**O**_**7**_**)**_**3**_**(PO**_**4**_**)**_**2**_

**DOI:** 10.1038/s41598-018-26597-w

**Published:** 2018-05-25

**Authors:** Navaratnarajah Kuganathan, Sashikesh Ganeshalingam, Alexander Chroneos

**Affiliations:** 1 Department of Materials, Imperial College London, London, SW7 2AZ United Kingdom; 2 Depratment of Chemistry, University of Jaffna, Sir Pon Ramanathan Road, Thirunelvely, Jaffna Sri Lanka; 3 Faculty of Engineering, Environment and Computing, Coventry University, Priory Street, Coventry, CV1 5FB United Kingdom

## Abstract

Layered Li_9_V_3_(P_2_O_7_)_3_(PO_4_)_2_ has attracted considerable interest as a novel cathode material for potential use in rechargeable lithium batteries. The defect chemistry, doping behavior and lithium diffusion paths in Li_9_V_3_(P_2_O_7_)_3_(PO_4_)_2_ are investigated using atomistic scale simulations. Here we show that the activation energy for Li migration via the vacancy mechanism is 0.72 eV along the c-axis. Additionally, the most favourable intrinsic defect type is Li Frenkel (0.44 eV/defect) ensuring the formation of Li vacancies that are required for Li diffusion via the vacancy mechanism. The only other intrinsic defect mechanism that is close in energy is the formation of anti-site defect, in which Li and V ions exchange their positions (1.02 eV/defect) and this can play a role at higher temperatures. Considering the solution of tetravalent dopants it is calculated that they require considerable solution energies, however, the solution of GeO_2_ will reduce the activation energy of migration to 0.66 eV.

## Introduction

Lithium ion batteries are widely used as a dominant power source in consumer electronics and electric vehicles^1–5^. Batteries with high-power density needed for large scale applications require new class of electrode materials providing large quantities of Li ions together with low cost, environmentally benign and constituent elements being high abundance. Several promising cathode materials for Li ion batteries [e.g. LiMPO_4_ (M = Fe, Mn and Co)^6,7^, Li_2_MSiO_4_ (M = Fe, Mn and Co)^6–10^ LiFeBO_3_^11^, LiFeSO_4_F^12^, Li_2_Fe(SO_4_)_2_^13^, Li_2_FePO_4_F^14^, Li_2_FeP_2_O_7_^15^ and Li_2_MnO_3_]^16^ including “Li rich” materials such as Li_7_Mn(BO_3_)_3_^17^ and Li_5_FeO_4_^18^ and supercapacitors [e.g. Li_3_V(MoO_4_)_3_^19^ and Li_4_Ti_5_O_12_]^20^ have been reported in the literature. The search for new class of cathode materials is still being continued in order to improve the output potential and energy density in Li ion batteries.

Layered vanadium monodiphosphate Li_9_V_3_(P_2_O_7_)_3_(PO_4_)_2_ was synthesised by Kuang *et al*.^21^ and suggested as a promissing cathode material as it provides a high concentration of Li^+^ ions (almost six Li ions per formula unit) together with a theoretical capacity of 173.45 mAhg^−1^ *via* a double-electron reaction where V^3+^ is oxidised to V^5+^. Further experimental studies were explored in this material to improve electrochemical performance, electronic and ionic properties by mixing with Na, doping with Cr and coating with carbon^22–24^. Recently, Balasubramaniam *et al*.^25^ have reported a cost effective way of synthesis and discussed the influence of crystallite size and carbon coating on the electrochemical performance. Jain *et al*.^26^ studied experimentally and theoretically the voltage, stability, volume change and diffusivity in Li_9_V_3_(P_2_O_7_)_3_(PO_4_)_2_. In the literature, there are no further theoretical studies detailing defect process, Li diffusion and dopants.

Static atomic scale modeling techniques based on the interatomic potentials are powerful tools to provide detailed information about the defect chemistry and Li ion migration pathways together with the activation barrier providing complementary information to experiment. In the present study, well-established atomistic modeling techniques are used to carry out a detailed survey of the relative energetics of the formation of intrinsic defects, solution of tetravalent dopants and the possible pathways for lithium ion conduction in Li_9_V_3_(P_2_O_7_)_3_(PO_4_)_2_.

## Results and Discussion

### Li_9_V_3_(P_2_O_7_)_3_(PO_4_)_2_ structure

**Figure 1:**
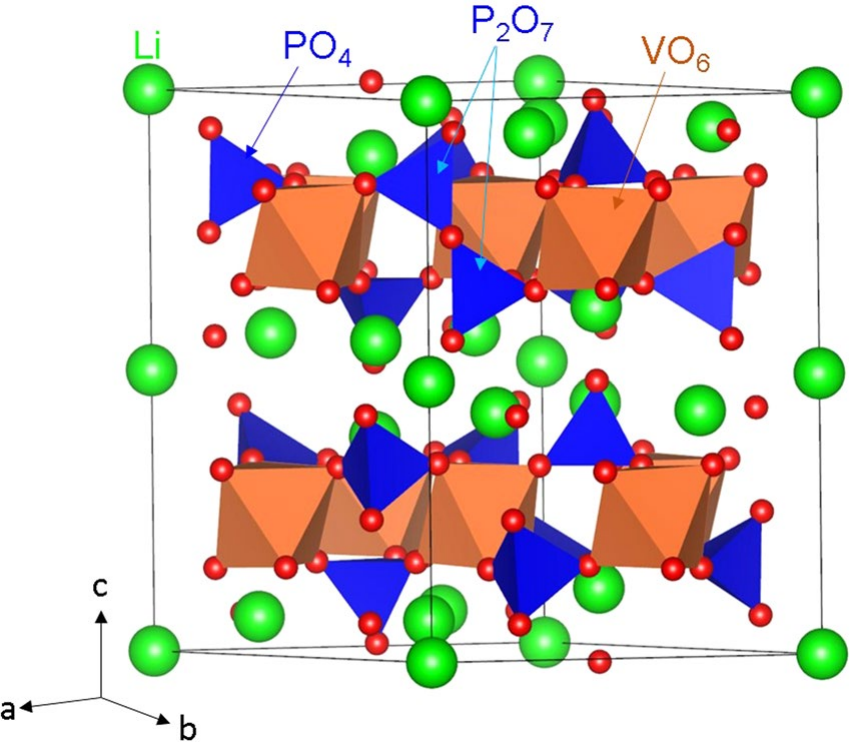
Crystal structure of Li_9_V_3_(P_2_O_7_)_3_(PO_4_)_2_ (space group P $\bar{3}$ C 1).

Crystal structure of Li_9_V_3_(P_2_O_7_)_3_(PO_4_)_2_ exhibits a layered trigonal crystallographic structure with space group P $\bar{3}$ C 1 (lattice parameters a = b = 9.728 Å, c = 13.591 Å, α = β = 90° and γ = 120°) as reported by Kuang *et al*.^23^ Fig. 1 shows this structure and the chemical environments of V (forming a octahedron with six O atoms) and P (forming a tetrahedron with four O atoms). Alternative anion and cation layers are present along the c direction and the anion layers contain V_3_(P_2_O_7_)_3_ (PO_4_)_2_ groups. The starting point for the present study was to reproduce the experimentally observed trigonal crystal structure to enable an assessment of the quality and efficacy of the classical pair potentials (refer to Table S1 in the supplementary information for the potentials parameters used and method section for the detailed description of the methodology) used in this study. The calculated equilibrium lattice constants (tabulated in Table 1) are in excellent agreement with experiment.

**Table 1:** Calculated and Experimental Structural Parameters for Orthorhombic (P $\bar{3}$ C 1) Li_9_V_3_(P_2_O_7_)_3_(PO_4_)_2_.

Parameter	Calc	Expt^19^	|∆|(%)
a (Å)	9.6714	9.7280	0.58
b (Å)	9.6714	9.7280	0.58
c (Å)	13.7659	13.5910	1.29
α = β (°)	90.0	90.0	0.00
γ (°)	120.0	120.0	0.00

### Intrinsic defect processes

To understand the electrochemical behavior of an electrode material, intrinsic defect processes are crucial. A series of isolated point defect (vacancy and interstitial) energies were calculated, which were combined to determine the formation energies for Frenkel and Schottky-type defects in Li_9_V_3_(P_2_O_7_)_3_(PO_4_)_2_. The following equations represent the reactions involving these defects as written using Kröger-Vink notation^27^.

1 $${\rm{Li}}\,{\rm{Frenkel}}:{{\rm{Li}}}_{{\rm{Li}}}^{{\rm{X}}}\to \,{V}_{{\rm{Li}}}^{{\prime} }+{{\rm{Li}}}_{{\rm{i}}}^{\cdot }$$

2 $${\rm{O}}\,{\rm{Frenkel}}:{{\rm{O}}}_{{\rm{O}}}^{{\rm{X}}}\to {V}_{{\rm{O}}}^{\cdot \cdot }+{{\rm{O}}}_{{\rm{i}}}^{{\prime}{\prime} }$$

3 $${\rm{V}}\,{\rm{Frenkel}}:{V}_{{\rm{V}}}^{{\rm{X}}}\to {V}_{{\rm{V}}}^{{\prime}{\prime} }+{{\rm{V}}}_{{\rm{i}}}^{\cdot \cdot \cdot }$$

4 $$\begin{array}{rl}{\rm{Schottky}}: & 9{{\rm{Li}}}_{\mathrm{Li}\,}^{{\rm{X}}}+3\,{{\rm{V}}}_{{\rm{V}}}^{X\,}+8{{\rm{P}}}_{{\rm{P}}}^{X\,}+29\,{{\rm{O}}}_{{\rm{O}}}^{{\rm{X}}}\to 9\,{V}_{{\rm{Li}}}^{{\prime} }+3\,{V}_{{\rm{V}}}^{{\prime}{\prime}{\prime} }+8\,{V}_{{\rm{P}}}^{{\prime}{\prime}{\prime}{\prime}{\prime} }\\  & +29\,{V}_{{\rm{O}}}^{\cdot \cdot }+{{\rm{Li}}}_{9}{{\rm{V}}}_{3}{({{\rm{P}}}_{2}{{\rm{O}}}_{4})}_{3}{({{\rm{PO}}}_{4})}_{2}\end{array}$$

5 $${{\rm{Li}}}_{2}{\rm{O}}\,{\rm{Schottky}}:2{{\rm{Li}}}_{{\rm{Li}}}^{{\rm{X}}}+{{\rm{O}}}_{{\rm{O}}}^{X\,}\to {\rm{2}}{V}_{{\rm{Li}}}^{{\prime} }+{V}_{{\rm{O}}}^{\cdot \cdot }+{{\rm{Li}}}_{2}{\rm{O}}$$

6 $${\rm{Li}}/{\rm{V}}\,\mathrm{antisite}\,\,({\rm{isolated}}):{{\rm{Li}}}_{{\rm{Li}}}^{{\rm{X}}}+{V}_{{\rm{V}}}^{X\,}\to {{\rm{Li}}}_{{\rm{V}}}^{{\prime}{\prime} }+{{\rm{V}}}_{{\rm{Li}}}^{\cdot \cdot }$$

7 $$\mathrm{Li}/V\,\mathrm{antisite}\,({\rm{cluster}}):{{\rm{Li}}}_{{\rm{Li}}}^{{\rm{X}}}+{\,V}_{{\rm{V}}}^{{\rm{X}}}\to {\{{{\rm{Li}}}_{{\rm{V}}}^{{\prime}{\prime} }:{{\rm{V}}}_{{\rm{Li}}}^{\cdot \cdot }\}}^{{\rm{X}}}$$

**Figure 2:**
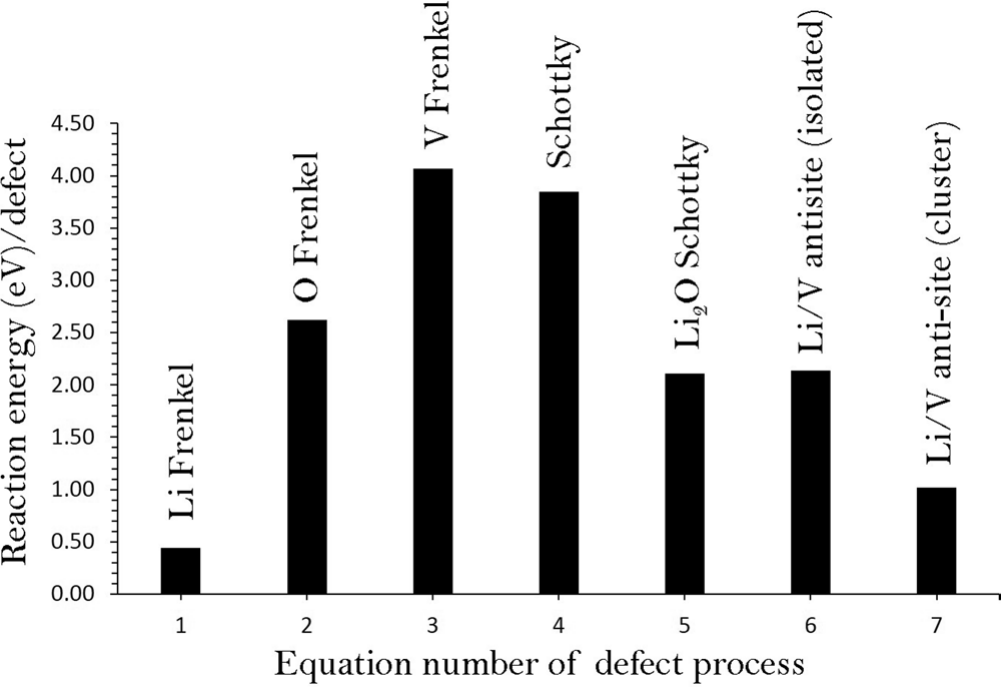
Energetics of intrinsic defect process in tetragonal Li_9_V_3_(P_2_O_7_)_3_ (PO_4_)_2_.

The reaction energies for these intrinsic defect processes are reported in Fig. 2 and Table S2. The most favorable intrinsic disorder is Li Frenkel and the formation of other Frenkel and Schottky defects is unfavourable. The second most favorable defect process is calculated to be anti-site. This indicates that there will be a small percentage of Li on V sites (${{\rm{Li}}}_{{\rm{V}}}^{^{\prime\prime} }$) and V on Li sites (${{\rm{V}}}_{{\rm{Li}}}^{\cdot \cdot })$ particularly at higher temperatures. It should be noted that this defect has been observed in a variety of Li ion battery materials during cycling^8,28–32^. The formation enthalpy of Li_2_O via the Li_2_O Schottky-like reaction (relation 5) is a processes that requires an energy of 2.11 eV per defect (refer to Table S2). This is a process that can lead to further $\,{V}_{Li}^{\text{'}}$ and ${V}_{O}^{\bullet \bullet }$ however at elevated temperatures.

### Lithium ion-diffusion

**Figure 3:**
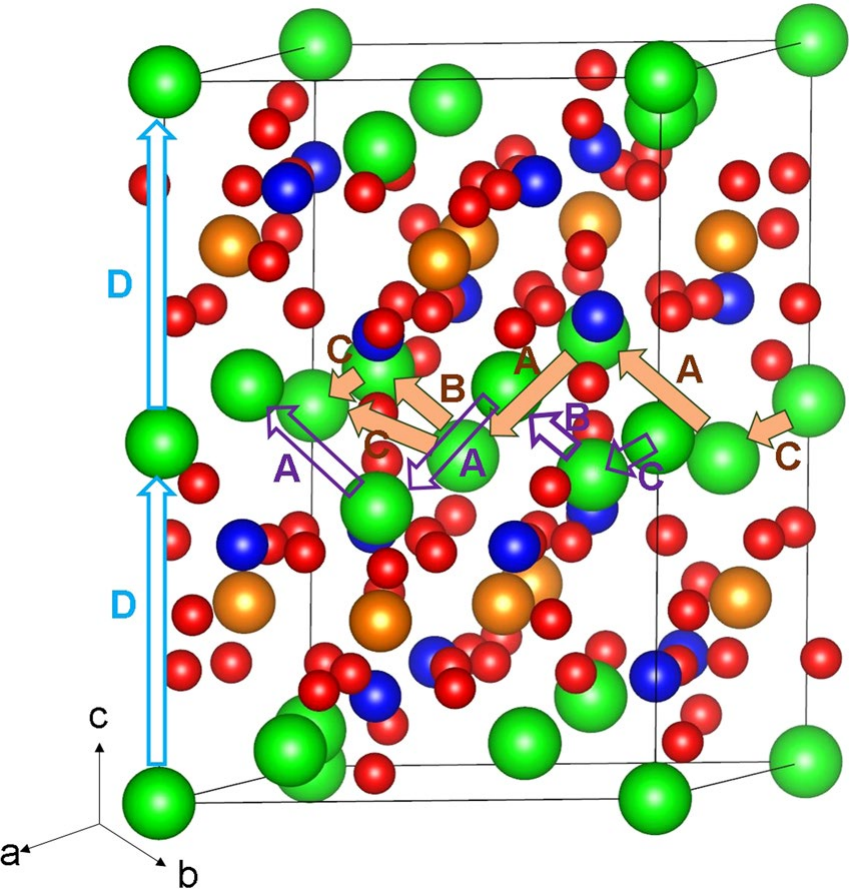
Possible long range lithium vacancy migration paths considered. Green, brown, blues and red colors correspond to Li, V, P and O atoms respectively.

**Figure 4:**
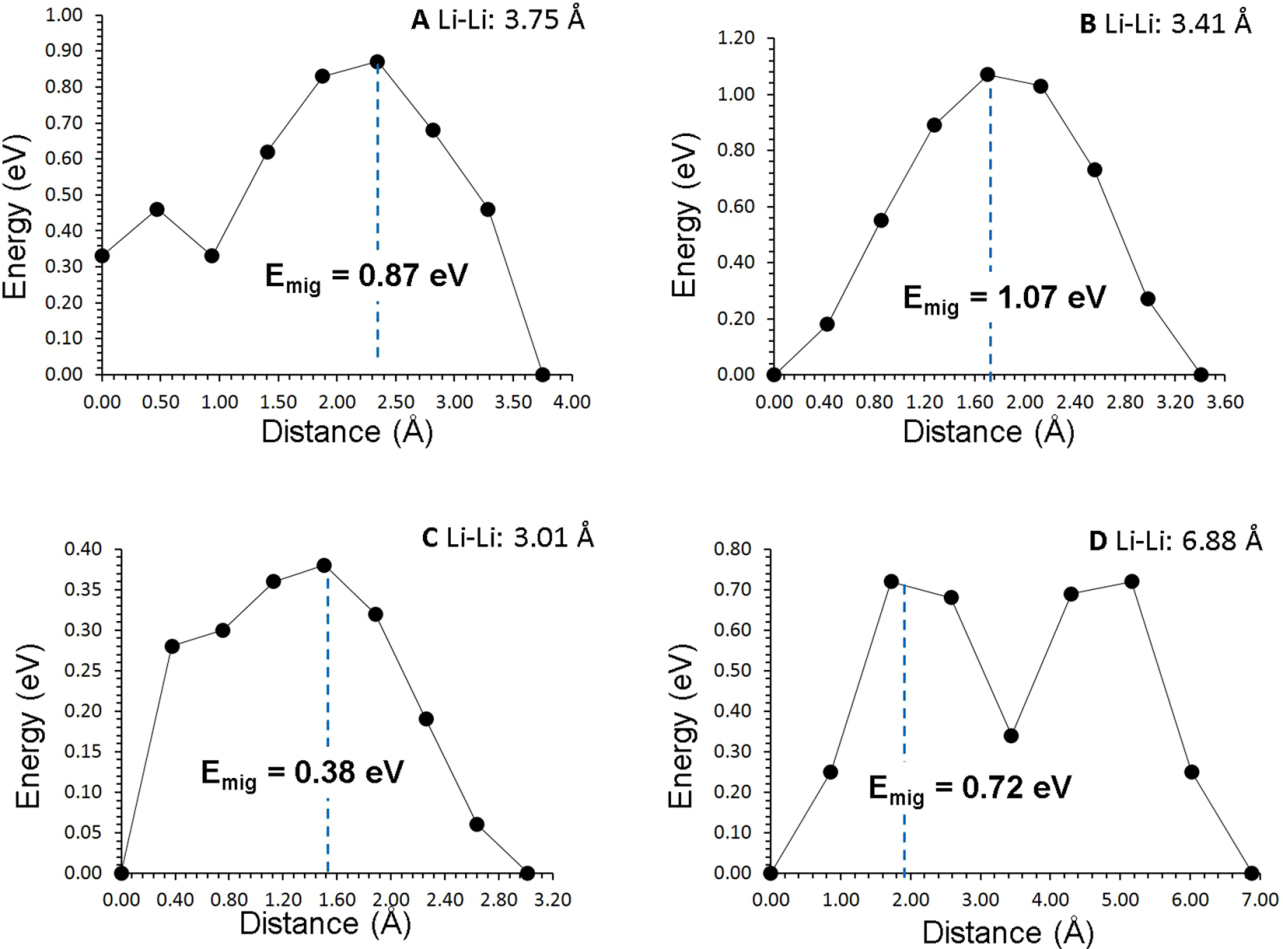
Four different energy profiles [as shown in Fig. 3] of Li vacancy hopping between two adjacent Li sites in Li_9_V_3_(P_2_O_7_)_3_(PO_4_)_2_.

The intrinsic lithium ion diffusion of Li_9_V_3_(P_2_O_7_)_3_(PO_4_)_2_ material is of crucial importance when assessing its use as a possible high-rate cathode material in lithium batteries. Using static atomistic simulation it is possible to examine various possible diffusion paths responsible for lihium ion conduction, which are often difficult to explore on the atomic scale by experiment alone. For the Li vacancy migration, we identified three lower energy long range paths connecting local Li hops (A, B C and D as shown in Fig. 3). There are two long range paths exhibit a zig-zag pattern along *ab* plane including a local Li hop with lower activation energy of migration of 0.38 eV but with overall activation energy of 1.07 eV (refer to Table 2 and Fig. 4). The third long range migration path along the c axis has identical Li hops with the activation energy of 0.72 eV. Thus this long range Li diffusion channel will have the overall activation energy of 0.72 eV in good agreement with the value of 0.74 eV reported by Jain *et al*.^26^. The activation energy of migration calculated along the *ab* plane is 1.30 eV^26^, which is in agreement with our calculated value of 1.07 eV. The difference in activation energy is due to description of ions in different methodologies. Here the activation energy of migration is defined as the position of the highest potential energy along the migration path. This indicates that long range diffusion is likely slow.

**Table 2:** Calculated Li-Li separations and activation energies for the lithium ion migration between two adjacent Li sites refer to Figs 3 and 4.

Migration path	Li-Li separation (Å)	Activation energy (eV)
A	3.75	0.87
B	3.41	1.07
C	3.01	0.38
D	6.88	0.72

### Tetravalent doping

The Li Frenkel is calculated to be only 0.44 eV/defect; however, an increase in the concentration of Li will further increase the applicability of Li_9_V_3_(P_2_O_7_)_3_(PO_4_)_2_ as a cathode material for rechargeable lithium batteries. A way to increase the content of intrinsic defects in oxides is by the solution of aliovalent dopants as it was previously demonstrated in CeO_2_ (for example ref.^33^ and references therein). Here we considered the solution of $R{O}_{2}$ (*R* = Ce, Zr, Ti, Si and Ge) via the following process (in Kröger-Vink notation):

8 $$2{{\rm{RO}}}_{2}+2{{\rm{V}}}_{{\rm{V}}}^{{\rm{X}}}+2{{\rm{Li}}}_{{\rm{Li}}}^{{\rm{X}}}\to 2{\,R}_{{\rm{V}}}^{\cdot }+2\,{V}_{Li}^{{\prime} }+\,{{\rm{V}}}_{2}{{\rm{O}}}_{3}+{{\rm{Li}}}_{2}{\rm{O}}$$

**Figure 5:**
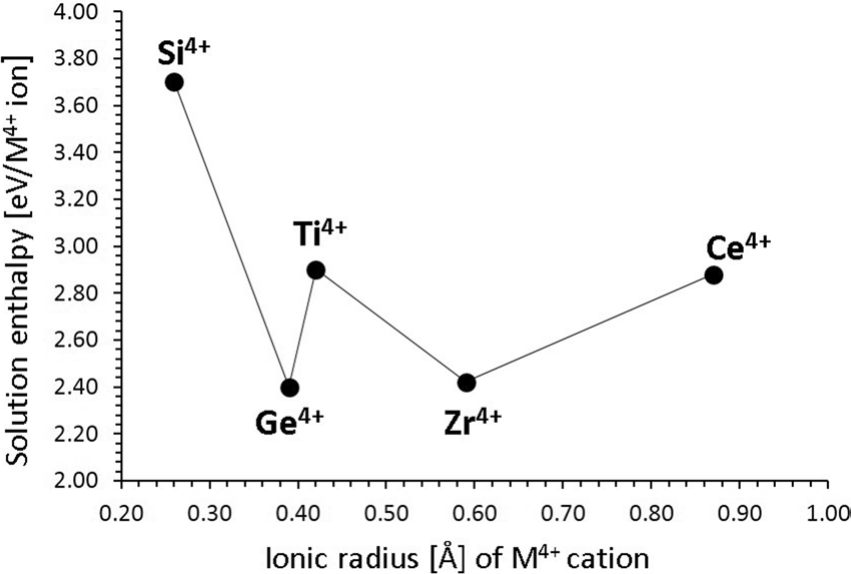
Enthalpy of solution of ${R}{{O}}_{2}$ (*R* = Ce, Zr, Ti, Si and Ge) with respect to the R^4+^ ionic radius in Li_9_V_3_(P_2_O_7_)_3_(PO_4_)_2_.

Figure 5 reports the solution energies of $R{O}_{2}$ and it can be observed that GeO_2_ and ZrO_2_ have the lowest ones 2.40 eV and 2.42 eV respectively. These solution energies are higher as compared to the Li Frenkel process nevertheless the solution of GeO_2_ or ZrO_2_ during synthesis should be examined experimentally as they can increase the Li vacancy concentration (via relation (8)).

**Figure 6:**
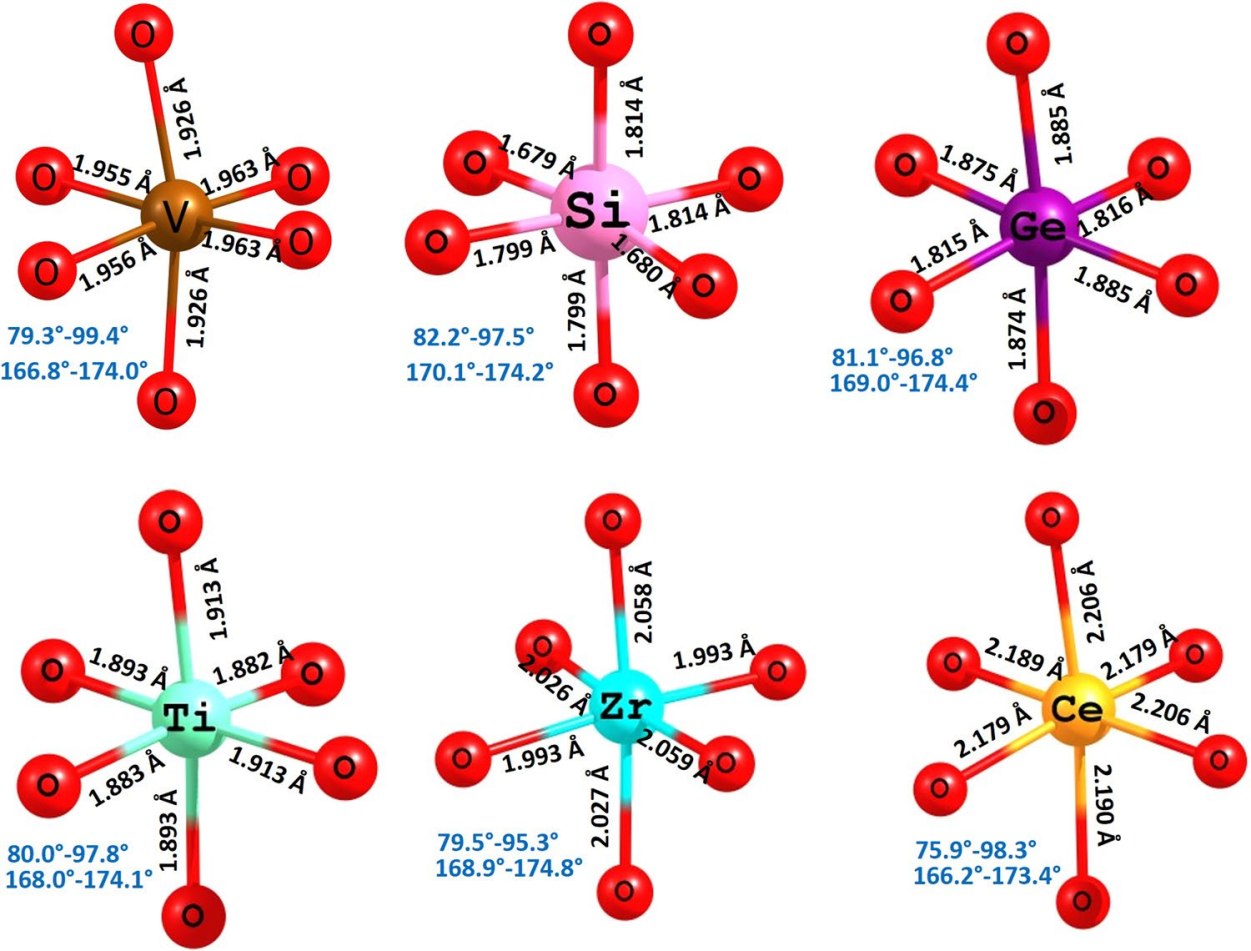
Octahedral VO_6_ unit in the relaxed structure of undoped Li_9_V_3_(P_2_O_7_)_3_(PO_4_)_2_. and the coordination formed by the dopants on the V site with neighbor oxygen.

Figure 6 depicts the local coordination (including bond lengths and angles) with oxygen of the dopants occupying the V site and for comparison the octahedral VO_6_ unit in the relaxed structure of undoped Li_9_V_3_(P_2_O_7_)_3_(PO_4_)_2_. The ionic radius of V^3+^ in octahedral coordination is 0.64 Å. The ionic radius of Si^4+^ is 0.38 Å smaller that that of V^3+^. In the SiO_6_ unit, there are two shorter bonds present compared to the other four Si-O bonds. This indicates that Si prefers SiO_4_ unit as observed in most silicates and this is reflected in the solution energy. The lowest solution energy is calculated for Ge. There are six Ge–O bonds present with approximately equal bond distances. The bond distances are ~0.1 Å shorter than the V–O bond lengths. Though Ge forms tetrahedral coordination in most of the complexes, the exact reason for the lowest solution energy should be due to other factors. The solution energy of Ti is ~0.40 eV higher than that of Ge. The second lowest solution energy is found for Zr. Zirconium normally forms octahedral *six*-*coordinate* complexes in their crystal structures and its ionic radius is closer to the ionic radius of V^3+^. This is reflected in the solution energy. In the relaxed structure of CeO_6_ unit, Ce–O bond lengths are approximately the same but ~0.20 Å longer than V–O bond lengths present in VO_6_ unit. Furthermore, the ionic radius of Ce^4+^ is 0.26 Å longer than V^3+^. Thus the solution energy is slightly high. However, the current solution energy values are still large and positive indicating that they are highly unfavourable.

**Figure 7:**
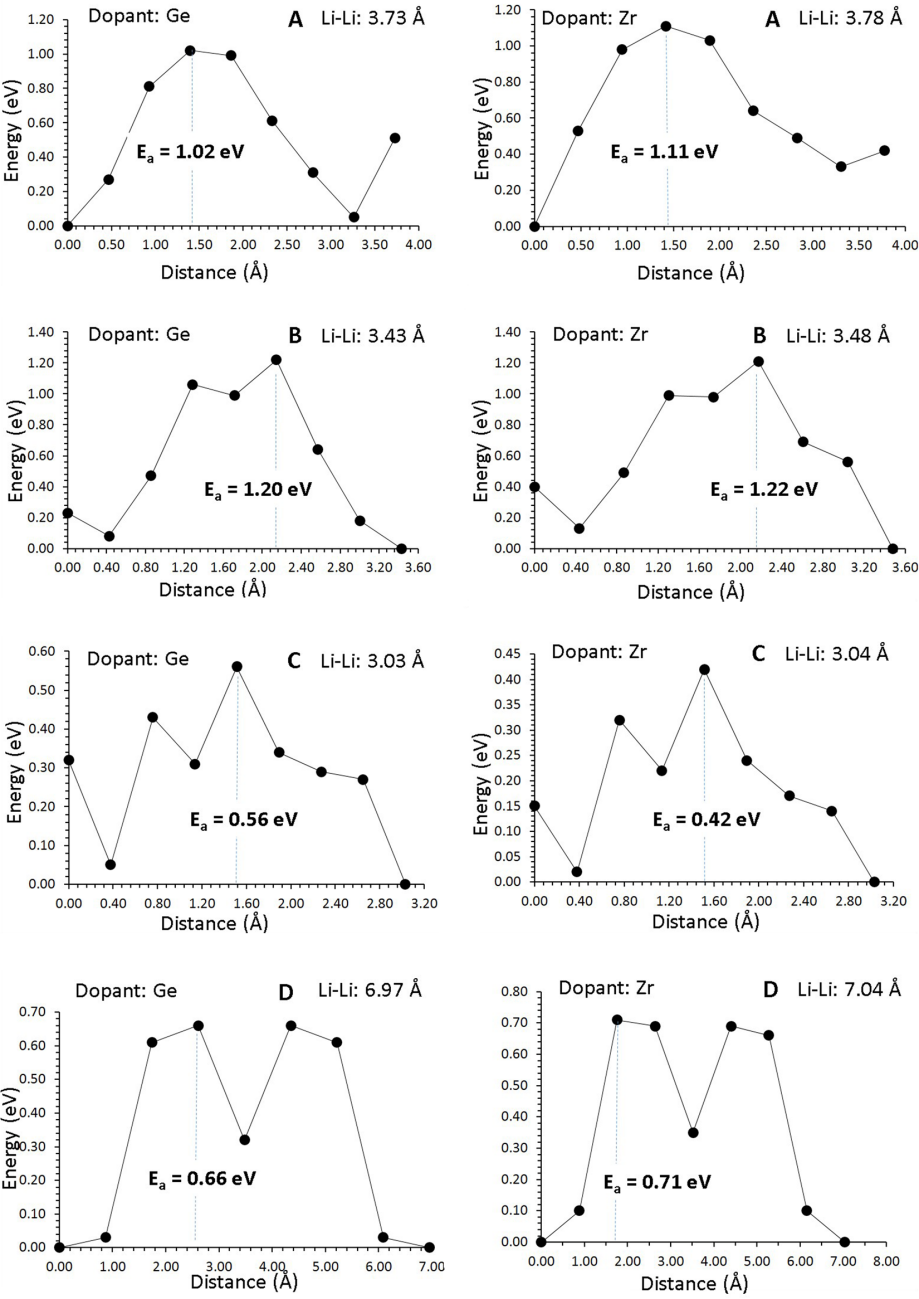
Energy profile diagrams for the Li vacancy hoping closer to the dopants (Ge and Zr) on the V site.

Introducing dopants in a lattice can also have an impact on the activation energies of migration. We present in Fig. 7 the energy profile diagrams for Li vacancy hoping closer to the Ge and Zr substitutionals as these are the lowest solution enthalpy dopants. The presence of the Ge and Zr substitutionals will increase the migration energy barriers of Li in the ab plane, but will reduce them in the c-axis mechanism where it matters as it is the lowest energy mechanism (refer to Figs 4 and 7). The activation energy of Li migration in the vicinity of Ge substitutionals is 0.66 eV that is 0.08 eV lower than in undoped Li_9_V_3_(P_2_O_7_)_3_(PO_4_)_2_.

### Summary

In the present study, the atomistic simulation techniques have been used to provide detailed insights into intrinsic defects, lithium ion mobility and tetravalent doping, which are relevant to the general electrochemical behavior of layered Li_9_V_3_(P_2_O_7_)_3_(PO_4_)_2_ as lithium battery cathodes. An advantage of this material is its low energy Li Frenkel (0.44 eV/defect). This will ensure that there will be considerable number of Li vacancies that are necessary as they act as vehicles for Li diffusion. We have considered the solution energies of $R{O}_{2}$ (*R* = Ce, Zr, Ti, Si and Ge) and calculated that GeO_2_ and ZrO_2_ have the lowest solution energies. These are far higher than the Li Frenkel process. At any rate if Li_9_V_3_(P_2_O_7_)_3_(PO_4_)_2_ doped with GeO_2_ is synthesized it will have a lower activation energy of migration by 0.08 eV along the c axis and a higher concentration of Li vacancies. The present defect engineering strategy can be employed to related systems to enhance the Li-ion diffusion.

### Methods

In order to calculate the energetics for the formation of intrinsic defects and possible Li ion diffusion pathways, the classical pair potential method as implemented in the GULP package^34^ was employed. This method is based on the classical Born model description of an ionic crystal lattice. All systems were treated as crystalline solids with interactions between ions consisting of the long-range attractions and short-range repulsive forces representing electron-electron repulsion and van der Waals interactions. The short range interactions were modelled using Buckingham potentials (refer to Table S1). Simulation boxes and the corresponding atom positions were relaxed using the Broyden-Fletcher-Goldfarb-Shanno (BFGS) algorithm^35^. The Mott-Littleton method^36^ was used to investigate the lattice relaxation about point defects and the migrating ions. It divides the crystal lattice into two concentric spherical regions, where the ions within the inner spherical region (on the order of >700 ions) immediately surrounding the defect relaxed explicitly. Li ion diffusion was calculated considering two adjacent vacancy sites as initial and final configurations. Seven interstitial Li ions were considered in a direct linear route and they were fixed while all other ions were free to relax. The local maximum energy along this diffusion path is calculated and reported as activation energy of migration. As the present model assumes a full charge ionic model with the calculations corresponding to the dilute limit the defect enthalpies will be overestimated, however, relative energies and trends will be consistent.

## Electronic supplementary material

Supplementary information
