# Supplementary material for: Defects, Dopants and Lithium Mobility in Li9V3(P2O7)3(PO4)2
Source: Sci Rep. 2018 May 25;8:8140. doi: 10.1038/s41598-018-26597-w (PMC5970228; doi:10.1038/s41598-018-26597-w)
Supplement: Supplementary file 1 — Supplementary information [file 41598_2018_26597_MOESM1_ESM.docx]

**Supporting Information**

**Defects, Dopants and Lithium Mobility in Li_9_V_3_(P_2_O_7_)_3_(PO_4_)_2_**

Navaratnarajah Kuganathan^1^, Sashikesh Ganeshalingam^2^, Alexander Chroneos^1,3^

*^1^Department of Materials, Imperial College London, London, SW7 2AZ, United Kingdom*

*^2^Depratment of Chemistry, University of Jaffna, Sir Pon Ramanathan Road, Thirunelvely, Jaffna, Srilanka*

*^3^Faculty of Engineering, Environment and Computing, Coventry University, Priory Street, Coventry CV1 5FB, United Kingdom*

**Table S1**. Interatomic potential parameters used in the atomistic simulations of Li_9_V_3_(P_2_O_7_)_3_(PO_4_)_2_.

(a) Two-body [Φ*_ij_* (*r_ij_*) = *A_ij_* exp (− *r_ij_* /*ρ_ij_*) − *C_ij_ / r_ij_*^6^]

| Interaction | *A* (eV) | *ρ* (Å) | *C* (eV·Å^6^) | Y (e) | K (eV·Å^-2^) |
| --- | --- | --- | --- | --- | --- |
| Li^+^–O^2−^ | 632.1018 | 0.2906 | 0.00 | 1.000 | 99999 |
| V^3+^–O^2−^ | 1410.82 | 0.3117 | 0.00 | 2.04 | 196.30 |
| P^5+^–O^2−^ | 897.2648 | 0.3577 | 0.00 | 5.000 | 99999 |
| O^2−^–O^2−^ | 22764.30 | 0.1490 | 27.89 | –2.86 | 74.92 |

(b) Three-body [Φ*_ijk_* = ½ *K_ijk_* (*θ* −*θ*_0_)^2^]

| Bonds | *k* (eV·rad^-2^) | θ_0_ (deg) |
| --- | --- | --- |
| O^2−^–P^5+^–O^2−^ | 1.322626 | 109.47 |

**Table S2.** Energetics of intrinsic defect process in Li_9_V_3_(P_2_O_7_)_3_(PO_4_)_2_.

| Defect process/equation | Reaction energy/eV | Reaction energy per defect/eV |
| --- | --- | --- |
| Li Frenkel /1 | 0.88 | 0.44 |
| O Frenkel /2 | 5.24 | 2.62 |
| V Frenkel /3 | 8.14 | 4.07 |
| Schottky /4 | 188.65 | 3.85 |
| Li_2_O Schottky/5 | 6.34 | 2.11 |
| Li/V antisite (isolated) /6 | 4.28 | 2.14 |
| Li/V antisite (cluster) /7 | 2.04 | 1.02 |
